# Perfluoropyridine: Discovery, Chemistry, and Applications in Polymers and Material Science

**DOI:** 10.3390/molecules27051616

**Published:** 2022-02-28

**Authors:** Ritesh Gautam, Ian Geniza, Scott T. Iacono, Chadron M. Friesen, Abby R. Jennings

**Affiliations:** 1 Department of Chemistry, United States Air Force Academy, Colorado Springs, CO 80840, USA; c22ritesh.gautam@afacademy.af.edu (R.G.); c23ian.geniza@afacademy.af.edu (I.G.); scott.iacono@afacademy.af.edu (S.T.I.); 2 Department of Chemistry, Trinity Western University, 22500 University Drive, Langley, BC V2Y 1Y1, Canada; chad.friesen@twu.ca

**Keywords:** perfluoropyridine, nucleophilic aromatic substitution, fluoropolymers, organofluorine

## Abstract

Perfluoropyridine (PFPy) is an organofluorine compound that has been employed for a variety of applications, from straightforward chemical synthesis to more advanced functions, such as fluorinated networks and polymers. This can be directly attributed to the highly reactive nature of PFPy, especially towards nucleophilic aromatic substitution (SNAr). The aim of this review is to highlight the discovery and synthesis of PFPy, discuss its reactive nature towards SNAr, and to summarize known reports of the utilization and thermal analysis of PFPy containing fluoropolymers and fluorinated network materials.

## 1. Introduction

The fluorine atom exhibits many unique properties, including a small atomic radius, large electronegativity, and minimal polarizability [1]. Thus, when coupled with carbon in the form of a C-F bond, organofluorine compounds with highly sought after properties can be obtained [2]. Many of these fluorinated compounds display increased hydrophobicity, as well as enhanced chemical and oxidative resistance, and improved thermal stability [3]. Accordingly, organofluorine compounds have found applications in a range of areas, such as medical imaging [4,5], pharmaceuticals [6], and agrochemicals [7]. Furthermore, driven by the growing demand to develop novel high-performance materials, fluorinated compounds are often used as the building blocks of more complex chemical systems, such as fluoropolymers and fluorinated network materials [8].

There are a number of different organofluorine compounds that have been used as starting materials for more complex systems and polymers. Organofluorine compounds used for these materials can be derived directly from a commercially sourced fluorinated starting material or prepared in-house using selective or direct fluorination techniques [9,10,11]. The motivation for choosing one feedstock over another weighs heavily on the overall synthetic demand of the polymer or material, scalability, cost, and desired properties. With the growing variety of commercially available fluorinated starting materials, often times, researchers will utilize commercial sources versus turning to the more synthetically demanding routes of direct fluorination.

Perfluoropyridine, or PFPy, is one example of these fluorinated compounds that has been used for a wide variety of purposes, ranging from general synthetic reactions [12,13] to complex pharmaceuticals [14,15,16] and materials [17,18,19]. Although it can be prepared in house by employing the methods described below, PFPy is commercially available, and is considered to be an economically viable option in modern times. Furthermore, as discussed in the subsequent sections, PFPy is a very reactive molecule and can be used to develop more complex fluorinated starting materials. The goal of the present review is to highlight the history and synthetic routes used to obtain PFPy, provide an overview of its unique reactivity, and demonstrate its use in preparing fluorinated networks and polymers. 

## 2. Discovery and Synthesis of Perfluoropyridine

**Scheme 1:**
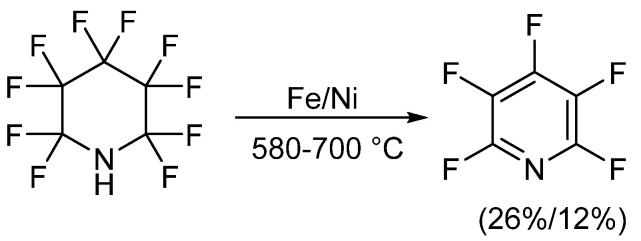
Synthesis of PFPy from perfluoropiperidine.

The first reported synthetic methods for obtaining PFPy occurred in the early 1960s and involved defluorination of perfluoropiperidine [20,21,22,23]. In these instances, perfluoropiperidine, which was synthesized electrochemically from pyridine and anhydrous hydrogen fluoride [24], was passed over a metal, either iron or nickel, at high temperatures. PFPy was then isolated through chromatographic methods. When iron was implemented, PFPy was obtained at a 26% yield. A slightly lower recovery, 12%, was found when defluorination was conducted using nickel (Scheme 1) [20,21,22].

**Scheme 2:**
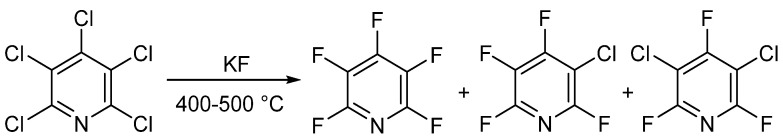
Synthesis of PFPy using anhydrous potassium fluoride.

In 1964 and 1965, two different groups—Chambers et al. [25,26,27] and Banks et al. [28,29]—published similar methods for the synthesis of PFPy. Here, the authors were able to prepare PFPy by heating pentachloropyridine in an autoclave with anhydrous potassium fluoride. Pentachloropyridine was prepared by reacting pyridine with phosphorous pentachloride [30]. In this instance, a mixture of products was obtained but could be separated by distillation (Scheme 2) [25,26,27,28,29]. 

The total amount of halogenated products was about 90%, and the ratio of products could be tuned by changing the temperature and reaction time. Under optimal conditions, PFPy could be obtained at a yield of 83% [29]. The method shown in Scheme 2 is still the gold standard for synthesizing PFPy commercially. 

**Scheme 3:**
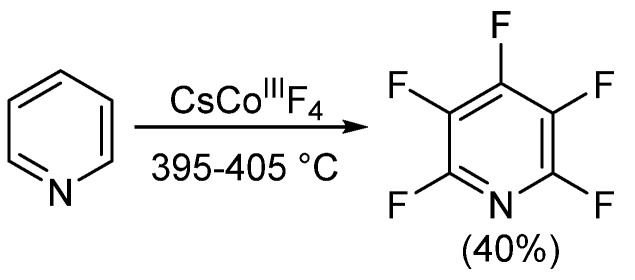
Synthesis of PFPy using cesium tetrafluorocobalatate (III).

Since the development of the commercial method for obtaining PFPy in the mid-1960s, little progress on obtaining PFPy by other means has been made. Nearly 20 years later, Banks et al. obtained trace quantities of PFPy by co-pyrolyzing pentafluoro(trychloromethy)benzene and 4-(dichloroamino)tetrafluoropyridine under nitrogen [31]. The same year, Coe and Sleigh pyrolyzed a number of pyrrolidines in the presence of iron [32]. This reaction produced a mixture of products, and similar to the report by Banks [31], PFPy was obtained in very low quantities (<12%). Later, in 1982, Plevey and co-workers were able to obtain PFPy at a yield of 40% by fluorinating pyridine with cesium tetrafluorocobalatate (III) [33]. Despite this, they were also met with challenges and encountered a point of diminishing returns on the yield when the reaction was attempted above a 5 g scale (Scheme 3) [33]. 

In a final example published in 2004 by Bardin et al., the authors were able to obtain PFPy by the dehalogenation of C_5_Cl_4_F_5_N in the presence of iron or zinc [34]. In all cases, a mixture of products was obtained and non-isolated yields, as determined by GLC analysis, remained average when compared to those obtained by the method presented in Scheme 2 (Table 1).

## 3. Chemistry of Perfluoropyridine

**Scheme 4:**
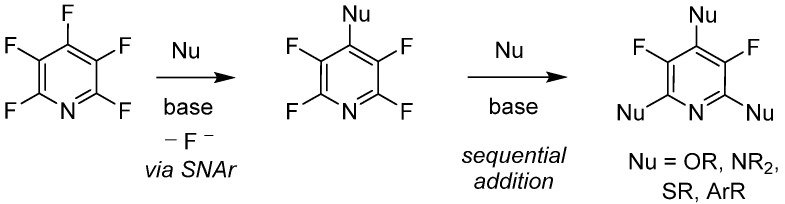
PFPy as a regio- and chemo-selective nucleophile.

Since its initial reported synthesis in the early 1960s, there have been over 600 publications in which PFPy has been utilized as a starting material or reagent [35]. With that, the chemical transformations of PFPy are well documented and some noteworthy methodologies include C-F bond activation [36], photoredox reactions [37,38], hydrodefluorination [39,40], and nucleophilic addition [41]. With the variety of readily available nucleophiles, the latter of these known chemical transformations has shown to be quite versatile. Furthermore, PFPy possesses unique regioselectivity, whereby stoichiometric addition to the 4-*para*-position is exclusive with a broad range of *O*-, *N*-, *S*, and *C*-nucleophiles under mild basic conditions. Sequential addition to the 2,6-*ortho*-positions can also be accomplished, dividing substitution at the 3,5-*meta*-positions (Scheme 4). 

This sequence is commonly accepted to proceed under classic nucleophilic aromatic substitution (SNAr). However, it has been recently debated whether these reactions proceed via a two-step process with a Meisenheimer intermediate or, rather, as a concerted reaction [42]. A concerted nucleophilic aromatic substitution reaction (CSNAr) mechanism supports the notion that electron-poor arenes cannot effectively stabilize a negative charge during Meisenheimer complex formation, but can favorably undergo substitution under mild conditions with a good leaving group, in this case fluoride. Furthermore, as detailed later in this review, PFPy is also chemo-selective with a large pool of di-nucleophiles, which has further expanded the utility of this molecule. A consolidated review of perfluoroaromatic chemistry, in particularly with PFPy, has highlighted comprehensive nucleophilic addition–substitution transformations demonstrating chemo- and regioselectivity [43]. In this section, some more recent or selected examples will demonstrate a broader utility of PFPy. 

**Scheme 5:**
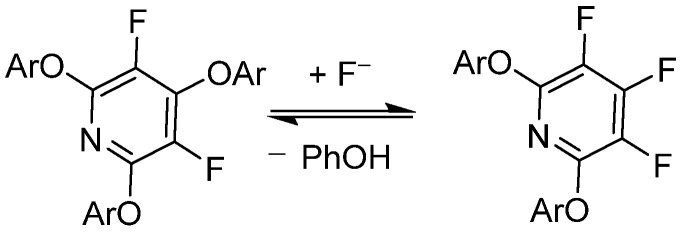
PFPy and its reversibility to complex products with phenols.

Vlasov et al. has demonstrated that PFPy undergoes reversible substitution with fluoride (F^−^) under basic conditions with good nucleophiles—in this case, phenols, as shown in Scheme 5 [44]. This work was further expanded to include a DFT modeling study of the reversibility effects of electron-rich/poor aryl ether substitutions at the 2,6-*ortho*-positions of PFPy [45]. Brittain et al. exploited these phenomena in order to utilize PFPy as a robust protecting group for a diverse pool of phenolics that exhibited mild cleavage/deprotection [12].

**Scheme 6:**
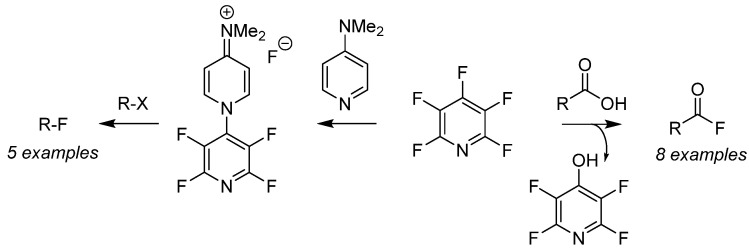
PFPyas a nucleophilic fluorinating agent.

Perfluoropyridine has been employed as a nucleophilic fluorination reagent for both alkyl halides (Scheme 6, *right-to-left*) [46] as well as deoxyfluorination of carboxylic acids (Scheme 6, *left-to-right*) [47] for the preparation of a versatile pool of substrates. Fluorination of organo-halides (R–X) is achieved after generation of the air-stable fluoride salt from the addition of PFPy and dimethylaminopyridine [46]. Furthermore, the in situ generation of acyl fluorides is readily accomplished by the nucleophilic addition of carboxylic acids with PFPy [47]. The ester intermediate then undergoes cleavage from unsequestered fluoride, producing phenol as a by-product and the desired acyl fluoride. This strategy was expanded to include the ‘one-pot’ preparation of amides and esters utilizing PFPy as a coupling agent. 

**Scheme 7:**
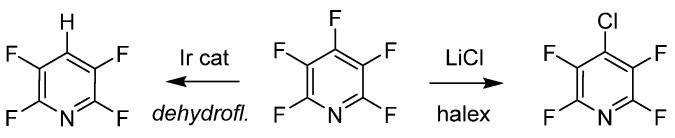
PFPy as a scaffold for selective hydrodefluorination.

The last examples of purposeful manipulation of PFPy involve site selective defluorination and halogen exchange (halex), as illustrated in Scheme 7 [48,49]. Both processes generate a regioselective substitution depending on various reaction conditions. Senaweera et al. demonstrated high photocatalytic turnover with low-Ir complex loading for the preparation of deyhdrofluorinated PFPy utilizing a flow reactor design, demonstrating the scalability of such useful intermediates [48]. The halex reverse-substitution was achieved in the 4-*para*-position of PFPy with LiCl, and additional exchanges on the ring with chlorine were achieved from a combination of kinetic and thermodynamic control [49]. 

## 4. Perfluoropyridine Used in Polymers and Materials

Since Teflon’s serendipitous discovery in 1938 by Roy J. Plunkett [50], the search for new fluorinated polymers and materials has continued. There are a considerable number of peer-reviewed reports highlighting this unique class of materials, their highly sought after properties, and advanced applications. In fact, just within the last five years alone, over 100 review articles and book chapters have been published on the topic of fluorinated materials and polymers [35]. The driving force for this is the continued demand for new and improved materials for advanced applications. Adding to the properties observed with simple organofluorine compounds, fluoropolymers and materials are also endowed with having low dielectric constants, refractive indexes, surface energy, flammability, and water absorptivity [51]. Furthermore, these materials are often more mechanically durable and morphologically diverse [52]. With such robust attributes, this unique class of materials has found use in a variety of specialized areas including the aerospace and automotive industries, advanced surfaces and coatings, medical devices, structural components, and many more [53,54]. 

Synthetic methods to obtain fluoropolymers and networks vary widely. As with simple organofluorine molecules, a fluoropolymer or fluorinated material can be achieved by direct fluorination methods [55] or by using a fluorinated monomer or building block [11]. With PFPy’s unique and high reactivity, it has been utilized in both routes—to fluorinate an existing polymer or material and to prepare more diverse fluorinated building blocks. The use of PFPy in fluorinated networks and polymers will be discussed below. 

**Scheme 8:**
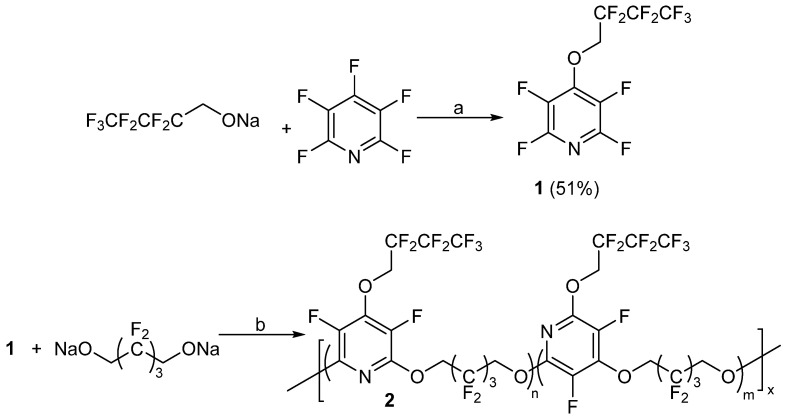
Synthesis of polymeric precursor **1** and homo/copolymer **2**. Conditions: a = DMF, 15 °C for 30 min then 45 °C for 1 h; b = THF/TMS, 15 °C for 1 h then 50 °C for 3.5 h.

One of the first known reports of using PFPy for the synthesis of polymeric materials was presented in the late 1970s by Johncock, Hewins, and Cunliffe [56]. With the implementation of PFPy in the polymers, the authors’ objective was to obtain fluoropolymers that exhibited enhanced structural and oxidative stability, low glass transition temperatures (*T*_g_s), and elastomeric properties, making the fluoropolymers appropriate materials for applications in the aerospace industry. With the knowledge that PFPy is reactive towards nucleophilic substitution and that it exhibits bifunctionality, the authors employed the Williamson Ether synthetic method to prepare new PFPy-based fluoropolymers. In one example, the authors initially reacted PFPy with the sodium salt of 2,2,3,3,4,4,4-heptafluoro-1-butanol to obtain a polymeric precursor, **1**. Precursor **1** was further reacted with the sodium salt of 2,2,3,3,4,4-hexafluoro-1-5-pentandiol to obtain a PFPy-based polymer (**2**, Scheme 8) [56]. 

Upon purification of the sample by solvent fractionation and characterization by ^19^F NMR, the authors determined that the fluoropolymer obtained was a mixture of a homopolymer (*n* = 0, m = 1), which was the result of the expected nucleophilic addition at 4-position, and copolymer (*n*/m = 2), which was the result of the nucleophilic substitution reaction in the 4-position and the competing displacement reaction between the fluorinated butoxy group and the fluorine atoms in the 2- and 6-positions [57]. 

**Scheme 9:**
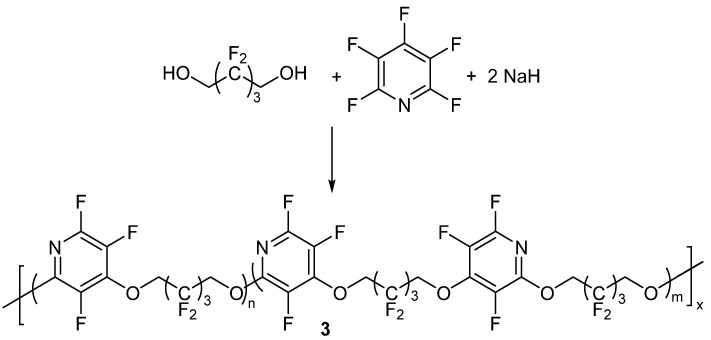
Synthesis of copolymer **3**. Conditions: THF/TMS, 5 °C for 2 h, RT for 16 h, then 50 °C for 5 h.

In another example, the authors also prepared a different copolymer by reacting the sodium salt of 2,2,3,3,4,4-hexafluoro-1-5-pentandiol with PFPy (Scheme 9) [56]. Under these conditions, the authors were able to obtain about 70% soluble polymer and roughly 6% of an insoluble gel. The gel was believed to be a network which resulted from the tri-substitution of the PFPy ring. The polymer fraction was isolated by solvent displacement. 

**Figure 1:**
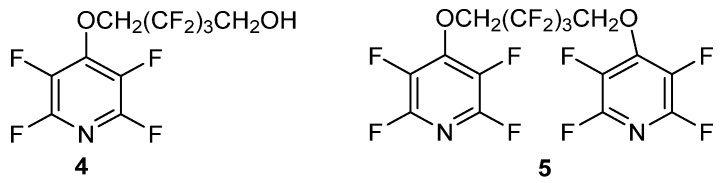
PFPy intermediates formed in the preparation of copolymer **3**.

The copolymer structure of **3** was determined by ^19^F NMR. Since the nucleophilic substitution of PFPy is more favorable at the 4-position than the 2 or 6-position [58,59], the authors concluded, that intermediates **4** and **5** (Figure 1) will likely form first. Intermediates **4** and **5** would then serve as the main building blocks of the fluoropolymer and support the structure of **3** [56]. 

The polymers shown in Scheme 8 and Scheme 9 were also characterized by differential scanning calorimetry (DSC), thermogravimetric analysis (TGA), and their viscosities were determined. Table 2 summarizes the data collected for **2** and **3** [56]. 

**Scheme 10:**
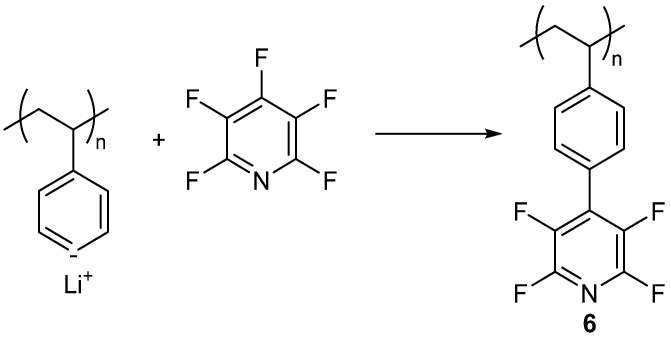
Synthesis of polymer **6**.

About 10 years later, the next known study of using PFPy in a polymer was reported by Banks and Tsilliopoulos [60]. In their example, the authors initially brominated and then lithiated linear polystyrene. The lithiated polystyrene was then reacted with PFPy to yield **6** (Scheme 10) [60]. Polymer **6** was fully fluorinated by treatment with F_2_/N_2_. No experimental or characterization data were provided for **6**, or the final, fully fluorinated material. This is the first study where PFPy is employed as a pendant group on the polymer vs. the backbone of the polymer/network. The PFPy was also not included in the original monomer but incorporated as the pendant group, post-polymerization. 

**Scheme 11:**
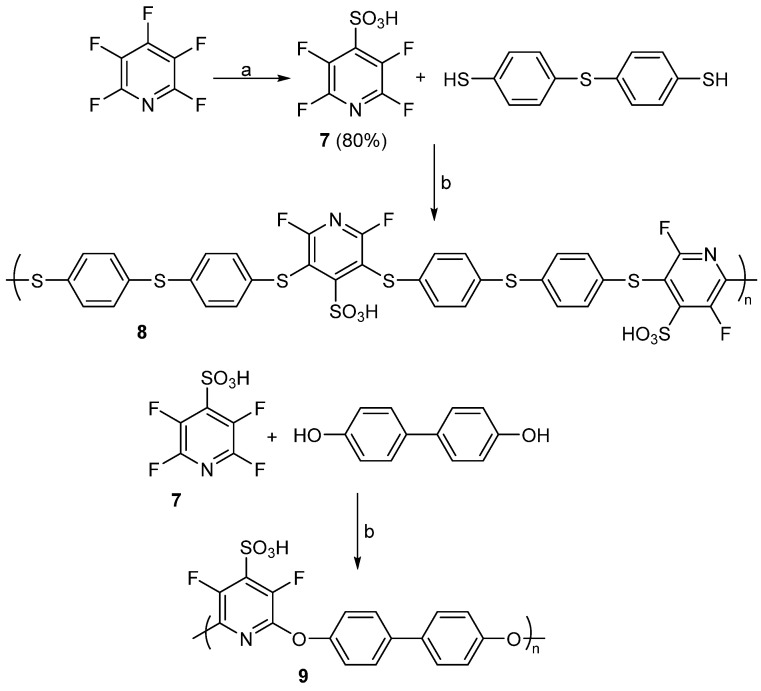
Synthesis of poly(arylene thioether) **8** and poly(arylene ether) **9**. Conditions: a = NaSH/H_2_O then HCOOH/H_2_O_2_; b = DMAc, 2 equiv. K_2_CO_3_, 100 °C for 24 h.

In a later study by Seyb and Kerres, the authors used PFPy to prepare sulfonated poly(arylene ethers) for potential applications in proton exchange membranes (PEMs) [61]. Initially, compound **7** was prepared by the nucleophilic thiolation of PFPy, followed by oxidation of the thiol to sulfonic acid. Next, **7** was reacted with 4.4′-thiobisbenzenethiol or 4,4′-dihydroxybiphenol to obtain a new poly(arylene thioether) or poly(arylene ether), respectively (**8** or **9**, Scheme 11) [61]. Characterization of polymer **8** and **9** by ^19^F NMR analysis indicated the structures shown. 

GPC analysis was used to determine the number average molecular weight (*M*_n_), weighted average molecular weight (*M*_w_), and dispersity (*Ɖ*) of **8** and **9**. Once characterized, the authors blended the polymers shown with polybenzimidazole (PBI)-Celazol^®^ in order to obtain PEMs that could be utilized in polymer electrolyte fuel cells. By incorporating **8** or **9** into the blend, the authors hoped to enhance the thermal stability and chemical resistance of the membrane towards H_3_PO_4_. Characterization of the polymers and blends by TGA-FTIR demonstrated that the onset of degradation for the sulfonic acid groups (T_SO_3___H_) ranged between 328 and 419 °C, whereas the degradation of the polymer backbone (T_backbone_) ranged between 332 and 461 °C. Additionally, **9** and its corresponding blend were more thermally stable than the **8** and its blend. Overall, the blends were more thermally stable than the polymers alone. In terms of oxidative stability, as determined by Fenton’s test, the authors concluded that future work to obtain higher molecular weight polymers was needed to fully optimize this parameter. The conductivity of the blends was also tested and compared to Nafion^®^. The films were stable up to 180 °C and also outperformed Nafion^®^. The results from the analyses performed by Seyb and Kerres are summarized in Table 3 [61]. 

**Scheme 12:**
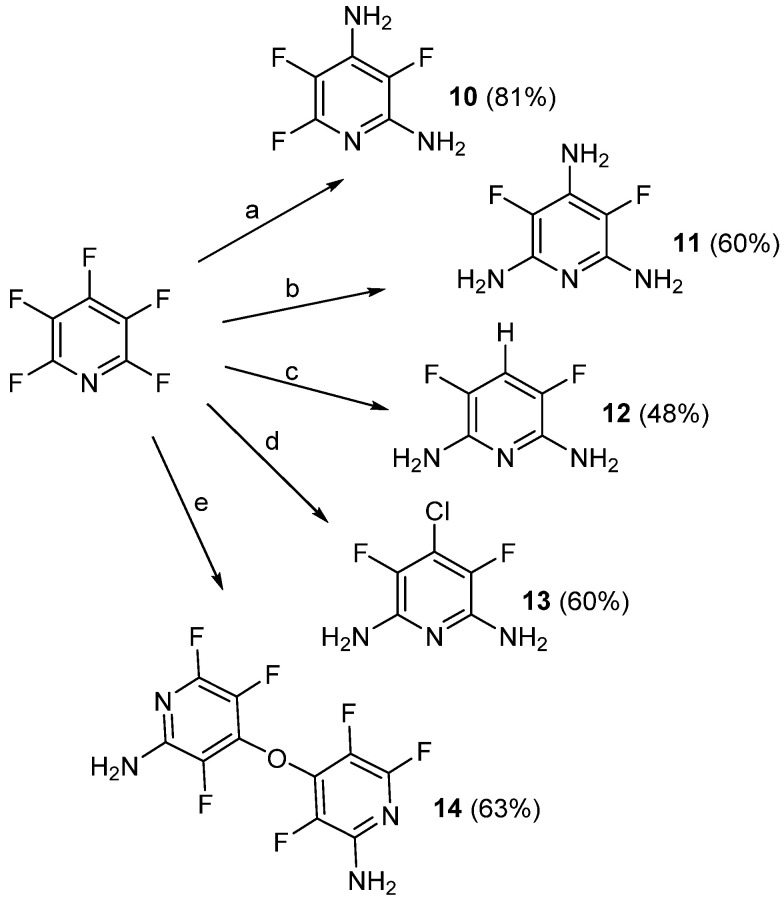
Fluorinated pyridylenediamine monomers prepared by Vaganova et al. Conditions: a = NH_3_ (ex), 100 °C, 15 h; b = NH_3_ (ex), 150 °C, 50 h; c = 1.1 equiv. Zn, 1.7 equiv. NaOH, H_2_O, 20 °C, 10 h, then NH_3_ (ex), 120 °C, 35 h; d = 1.5 equiv. CaCl_2_, dicyclohexano–18-crown-6, sulfolane, reflux, 5 h, then NH_3_ (ex), 110 °C, 10 h; e = tetrafluoro-4-pyridinoxide, sulfolane, reflux, 20 h, then NH_3_ (ex), 10–15 °C, 10 h.

Between 2007 and 2013, Vaganova et al. utilized PFPy to make a number of fluorinated pryidylenediamine monomers (**10**–**14**, Scheme 12) [62,63,64]. 

**Scheme 13:**
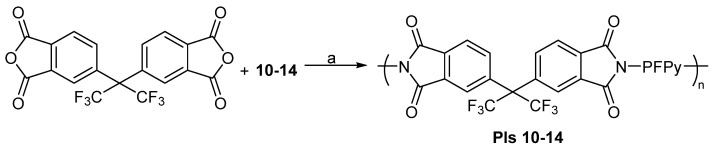
PIs prepared from fluorinated pyridylenediamine monomers, **10**–**14**. Conditions: a = stepped heating, BA melt.

These monomers were then used in a later study to prepare fluorinated polyimides (PIs) and co-polyimides (co-PIs), using one-step polycondensation reactions. The polymers were prepared for potential applications as materials for carbonized membranes [64]. For the PIs, a stepped heating was used and all polymerizations were carried out in benzoic acid (BA) melts (Scheme 13) [64]. The number average molecular weights of the PIs were determined by ^19^F NMR. 

**Scheme 14:**
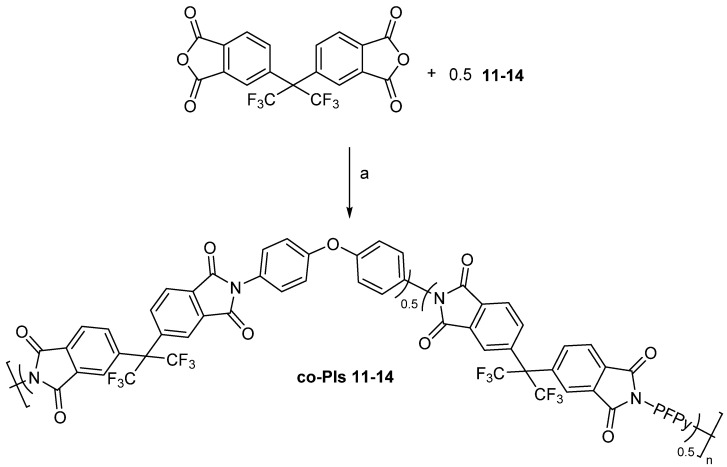
Co-PIs prepared from fluorinated pyridylenediamine monomers, **10**–**14**. Conditions: a = 140 °C, BA melt, then 0.5 equiv. oxydianiline, BA melt.

Similarly, the co-PIs were prepared from monomers **11**–**14** and 4,4′-oxydianiline in order to obtain higher molecular weight polymers that exhibit enhanced mechanical properties (Scheme 14) [63]. The molecular weights were not determined for the co-PIs, but the authors concluded that they had higher molecular weights than the PIs because these materials had increased viscosities. 

The PIs and co-PIs were thermally and thermo-oxidatively characterized using DSC and TGA analysis. The *T_g_*s of the PIs were found to be higher than the co-PIs. The lower *T_g_*s obtained for the co-PIs were attributed to the increased flexibility of the polymers as a result of the incorporation of 4,4′-oxydianiline. The polymeric materials demonstrated good thermal and oxidative stabilities, with most having T_5%_ ≥ 500 °C. Table 4 summarizes some of the characterization data obtained by Vaganova et al. [64]. Mechanical testing was performed using dynamic mechanical analysis (DMA) on films prepared from the co-PIs. These materials retained their storage modulus up to their *T_g_*s. The authors concluded that the co-PI films had good thermomechanical properties as a result of the 4,4′-oxydianiline; however, the PIs were not analyzed by DMA. 

The PIs were pyrolyzed under inert conditions and the chemical composition was analyzed at various temperatures in order to evaluate the polymers’ potential for use in carbonized materials. It was determined that the PIs underwent a selective and consecutive elimination of the different heteroatoms contained within and this could be utilized to tailor the properties of the nanostructured carbonized membranes formed from PIs **10**–**14** [64]. 

**Scheme 15:**
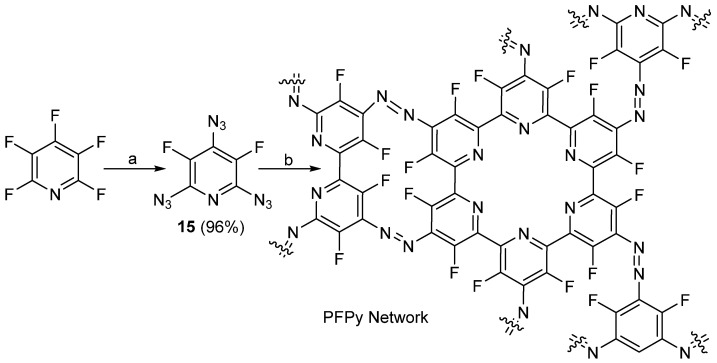
Thermal decomposition of 2,4,6-triazido-3,5-difluoroyridine, **15**. Conditions: a = DMSO, 3.2 equiv. NaN_3_, RT, 10 h; b = 160–172 °C.

In 2018, Nedel’ko et al. synthesized 3,5-difluoro-2-4-6-triazidopyridine using a previously published method, which involved the SNAr of PFPy with sodium azide [65]. The authors then studied the thermal decomposition of **15** using DSC. The degradation products were also characterized using IR spectroscopy [66]. Comparing the IR spectra of the degradation products of **15** with previously studied compounds (2,4,6-triazidopyridne, 2,4,6-triazidopyrimidine, and cyanuric triazide), the authors determined that because **15** lacks hydrogen atoms, its degradation mechanism favors the formation of a network of planar polyconjugated carbon–nitrogen porphyrin-like structures as a result of a complex chain polymerization (Scheme 15) [66]. 

**Scheme 16:**
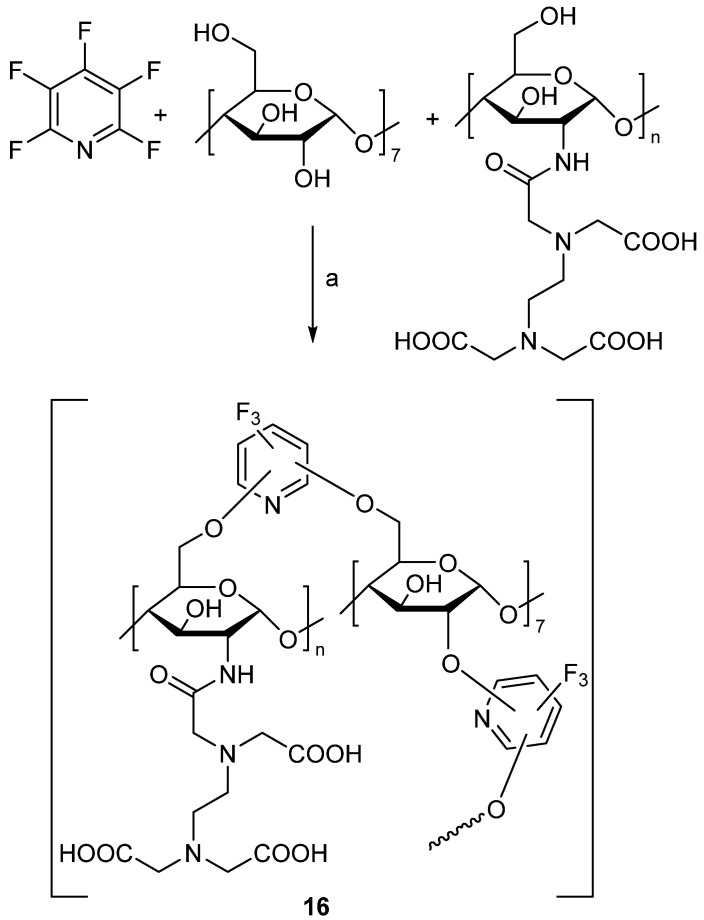
Synthesis of PFPy-functionalized porous β-CD polymers. Conditions: a = MeTHF:H_2_O, K_2_CO_3_, 80 °C, 48 h.

Additionally, in 2018, Yu and co-workers utilized PFPy to prepare porous β-cyclodextrin (β-CD) polymers for use in the removal of heavy metals and organic pollutants from water [67]. In their study, the authors utilized PFPy to join EDTA-modified chitosan with β-CD (or cellulose, sodium alginate, and alkali lignin) through the SNAr reaction of the hydroxyl groups of the biomolecules (Scheme 16) [67].

The resulting polymer, **16**, was characterized by FT-IR and solid state ^13^C NMR spectroscopy, which confirmed the incorporation of PFPy with the EDTA-modified chitosan and β-CD polymers. Further characterization by Brunauer–Emmett–Teller (BET) surface area analysis and scanning electron microscopy (SEM) indicated that **16** was porous and had a specific surface area of 47.8 m^2^/g, making it a promising material for an adsorbent. Energy dispersive X-ray analysis showed that **16** contained both nitrogen and fluorine, supporting the results observed by FT-IR and solid state ^13^C NMR spectroscopy. Adsorption studies were conducted with **16** and Pb (II), Ni (II), Cu (II), bisphenol A, trichlorophenol, and 6-bromo-2-napthol. The polymer was found to remove the contaminants with over 91% efficiency, whereas a sample consisting of cross-linked β-CD polymer only had a 40% efficiency under comparable conditions. Furthermore, **16** was found to be recyclable and, after five cycles, still maintained over 91% adsorption capacity [67].

**Scheme 17:**
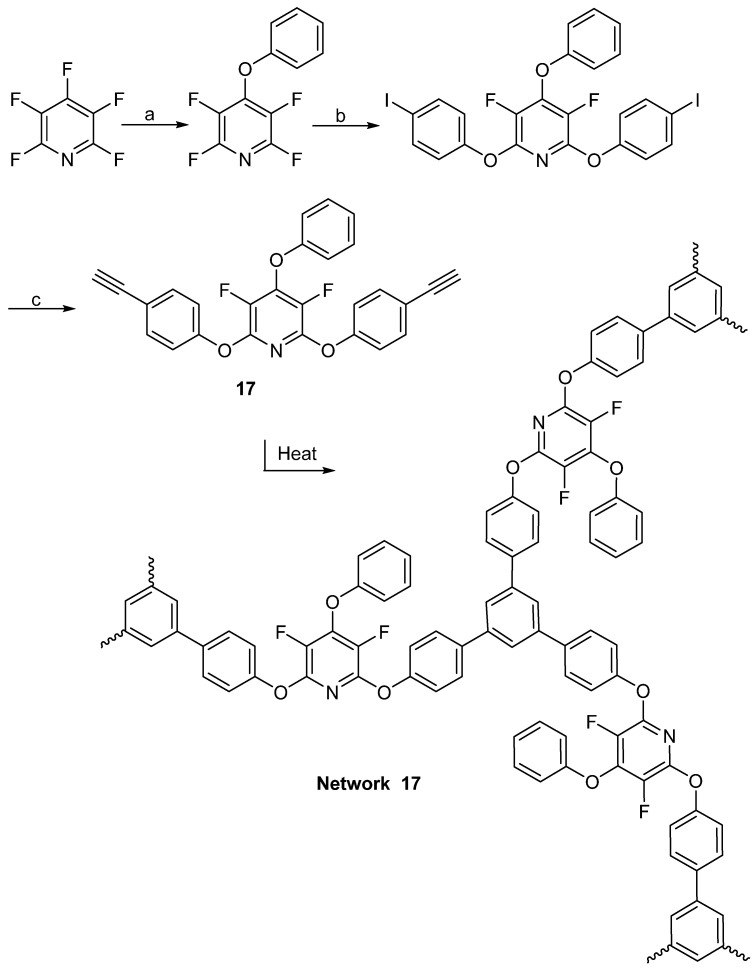
Preparation and network formation of **17**. Conditions: a = ArOH, DMF, K_2_CO_3_, RT, 16 h; b = 4-I-PhOH, Cs_2_CO_3_, DMF, 45 min; c = Pd/CuI, ethynyltrimethylsilane, and then TBAF.

In 2019, Corley et al. demonstrated [2 + 2 + 2] cyclopolymerizations of 2,6-bis(4-ethynylphenoxy)-3,5-difluoro-4-aryloxypyridine (**17**) for thermoset resins having intractable polyarylene networks [68]. All the polymeric products communicated in their work were derived from commercially sourced feed stocks of PFPy and functionalized phenols. To synthesize **17**, oxygen-based nucleophiles go through a selective SNAr reaction with PFPy specifically at the 4-postion using potassium carbonate. The additions at the 2 and 6-positions onto PFPy are then favorable with 4-iodo-phenol when using cesium carbonate in DMF. To complete the monomer (**17**), palladium and copper iodide are used to couple an ethyne onto the aromatic ring, by way of interacting TMS-ethyne with the iodo-bond on the aromatic ring. Thermal analysis indicated crosslinking of the monomer to form Network **17** (Scheme 17) [68].

**Figure 2:**
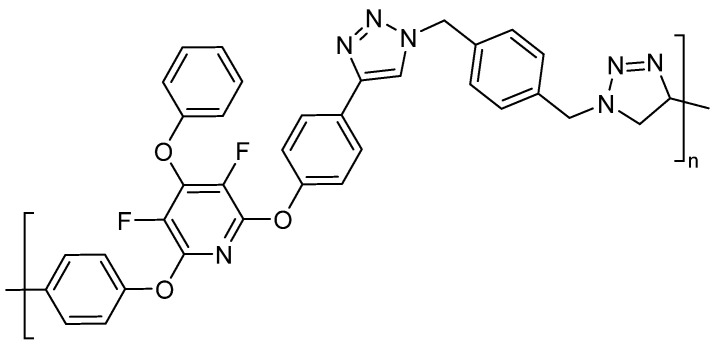
Polymer prepared from the cycloaddition of **17**. Conditions: DCM, 4-bis(azidomethyl)benzene, CuI, HOAc, DPEA, RT, 24 h.

Further cycloadditions were also considered and demonstrated. For example, 1,3-dipolar alkyne-azide “click” cycloaddition reactions were accomplished with the reaction of **17** with mono- or bis-(azidomethyl) benzene, generating a variety of polytriazole polymers (Figure 2) [68].

In all cases, these network materials showed excellent temperature resistance. The 2 + 2 + 2 cycloaddition networks exhibited thermal decomposition onset at 450 °C. The slow, near-linear weight loss of 45 wt% from 500 °C to 1000 °C (0.9 wt% °C − 1) under nitrogen is indicative of a highly dense network of carbonaceous material. A 20 wt% glassy carbon char was evident at 1000 °C. For the “click” cycloaddition polymer, the onset of degradation at 300 °C with linear decomposition to a glassy carbon char yield of 10 wt% is typical for polytriazoles with molecular weights between 2000 and 8000 g/mol. The authors also suggested that the high thermal stability indicated that the cycloadditions did not compromise the fluorinate pyridine core repeat unit [68].

**Scheme 18:**
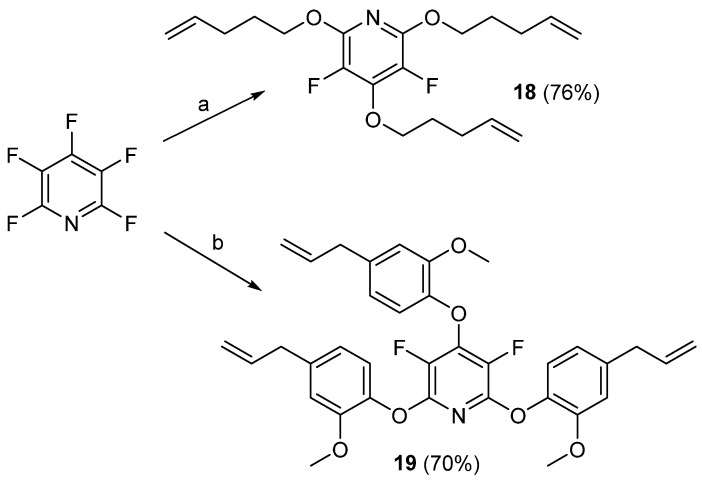
Synthesis of monomers **18** and **19**. Conditions: a = 4-penten-1-ol, DMF, Cs_2_CO_3_, 65 °C, 24 h; b = eugenol, DMF, Cs_2_CO_3_, 65 °C, 24 h.

In early 2020, Moore and co-workers prepared a new class of semifluorinated thiol-ene thermoset materials that were explored as aerospace sealants and coatings [69]. In this study, the authors developed two new trisubstituted alkene monomers, **18** and **19**, by reacting 4-penten-1-ol and eugenol with PFPy (Scheme 18) [69]. 

**Figure 3:**
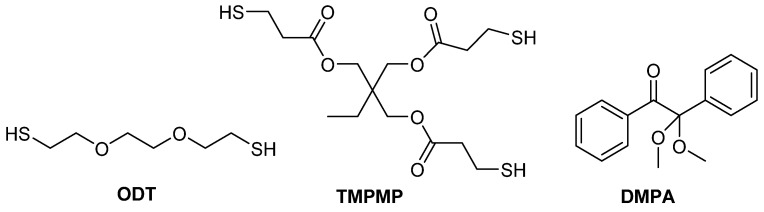
Multifunctional thiols and photo-initiator utilized to make thiol-ene thermosets.

Thiol-ene thermoset materials were synthesized using 3,6-dioxa-1,8-octan-edithiol (ODT) and trimethylolpropane tris(mercaptopropionate) (TMPMP) and the newly targeted alkene monomers. Formulations were mixed at a 1:1 thiol:ene ratio, with 1 wt% of 2,2-dimethoxy-2-phenylacetophe-none (DMPA) as the UV photo-initiator (Figure 3) [69].

The resulting polymeric systems based on these novel monomers demonstrated *T_g_*s ranging from −42 to 21 °C. The use of the trifunctional thiol results in a higher *T_g_* (i.e., TMPMP). Thermal degradation temperatures at 5% mass loss (*T*_d,5%_) spanned from 274 to 348 °C in nitrogen and char yields varied from 13.2 to 28.7% (see Table 5) [69].

This new class of semifluorinated thermoset materials with their measured properties have several potential applications within the aerospace industry, such as sealants and coatings, where stability and survivability at high temperatures in harsh environmental conditions are imperative [69].

**Scheme 19:**
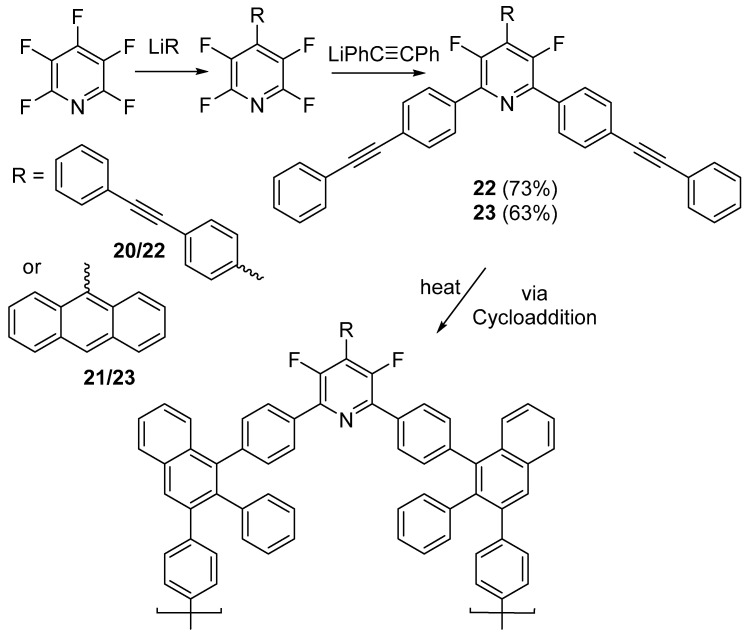
Synthesis and network formation of **22** and **23**.

At around the same time, Houck et al. reported the synthesis of two highly soluble high aromatic content (HAC) perflfluoropyridine-based thermosetting precursors [70]. Previously, to obtain polymers or materials that contain HAC, transition-metal-catalyzed coupling or harsh fluorination methods were required. However, utilizing the reactivity of PFPy towards SNAr, Houck et al. readily obtain monomers **22** and **23** in moderate yields. Initially, regioselective nucleophilic addition takes place with PFPy by first reacting it with lithiated 1,2-diphenylethyne or anthracene at the 4-position, to generate compounds **20** and **21**, respectively. To complete the monomers, further reactions are accomplished at both the 2 and 6-position by lithiated 1,2-diphenylethyne to generate monomers **22** and **23**, respectively (Scheme 19) [70]. 

Upon curing **22** and **23** at 341 and 372 °C (*T*_max_), respectively, both systems yielded insoluble cross-linked networks. The cured network of **22** displayed an onset of decomposition (*T*_d_) of 470 °C and a char yield of 51% at 900 °C, while the cured network of **23** displayed a *T*_d_ of 456 °C and a char yield of 81% at 900 °C (Table 6) [70].

The ease of thermal setting, combined with the formation of rigid cross-linked networks of highly densified polyarylenes exhibiting high decomposition temperatures and high char yields indicate these thermoset polymers are promising templates for nanomaterials in microelectronics and for use in high-temperature applications in the aerospace industry [70].

**Scheme 20:**
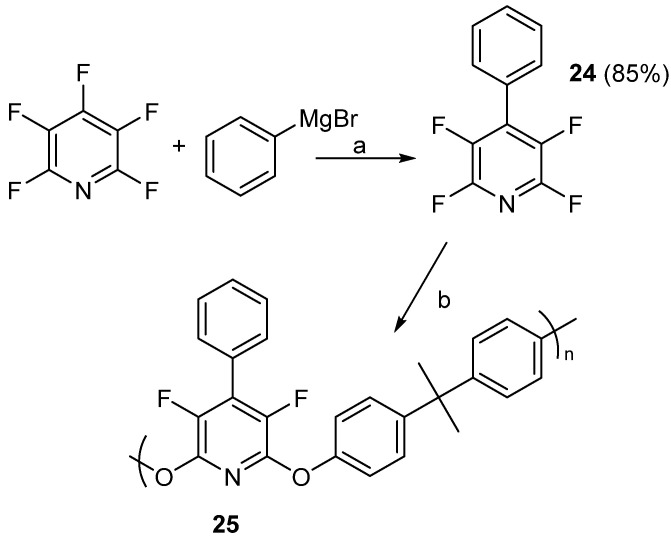
Step-growth polymers prepared from compound **24**. Conditions: a = THF, RT, 24 h; b = DMF, bisphenol-A, 0.3 equiv. NaH, 65 °C, 16 h.

In mid-2020, Eismeier and co-workers employed PFPy to prepare step-growth polymers and networks [71]. To prepare the step-growth polymers, compound **24** was prepared by SNAr reaction of PFPy with phenylmagnesium bromide [72]. This was then reacted with bisphenol A (BPA) to obtain the step-growth polymer, **24** (Scheme 20) [71]. ^19^F NMR indicated successful nucleophilic addition and formation of the desired polymer. End group analysis of **25** by ^1^H NMR indicated a molecular weight of 4000 g/mol.

**Scheme 21:**
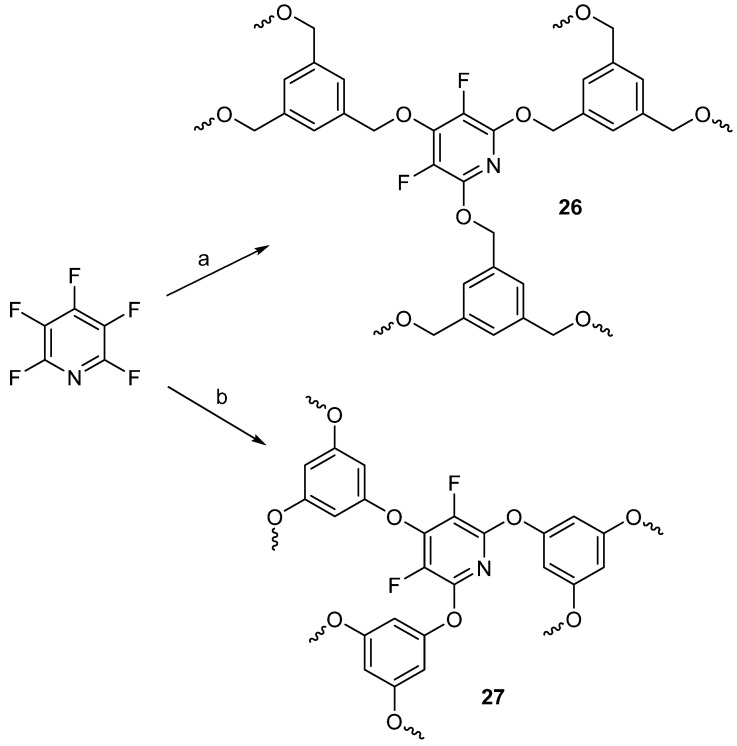
Network polymers prepared from PFPy. Conditions: a = 1,3,5-benzenetrimethanol, DMF, 3.3 equiv. NaH, 65 °C, 16 h; b = phloroglucinol, DMF, 3.3 equiv. NaH, 65 °C, 16 h.

Additionally, PFPy was reacted using similar conditions with 1,3,5-benzenetrimethanol or phloroglucinol to prepare fluorinated network materials, **26** and **27**, respectively (Scheme 21) [71].

Networks **26** and **27** were thermally characterized by DSC and TGA. The results from these studies are summarized in Table 7. The TGA analysis was performed under argon. Given the char yield of the networks and chemical composition of the materials, **25**–**27** could find potential applications for resins in the aerospace industry, low surface energy materials, and low dielectric constant materials [71].

**Scheme 22:**
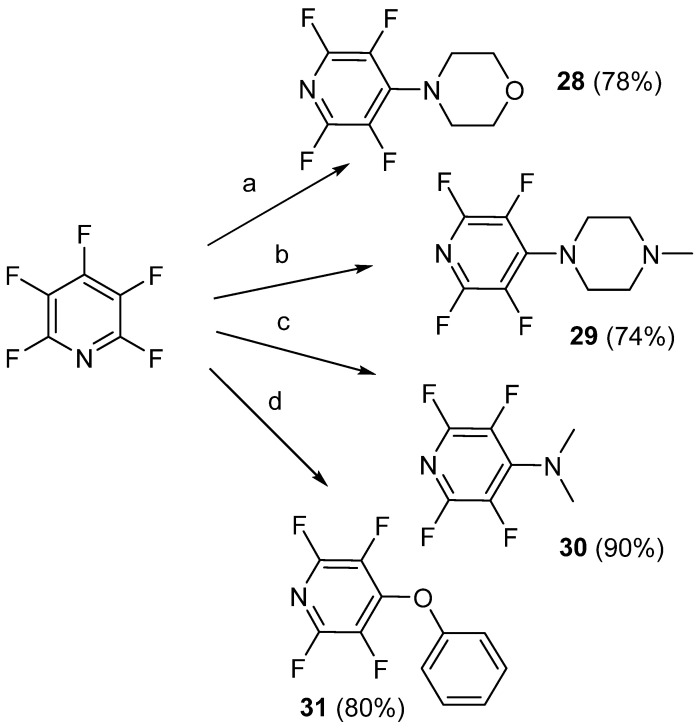
Synthesis of pyridinium-based monomers, **28**–**31**. Conditions: a = morpholine, NEt_3_, THF, RT, 48 h; b = N-methylpiperazine, NEt_3_, THF, RT, 48 h; c = dimethylamine, EtOH, 0 °C, 10 min; d = phenol, K_2_CO_3_, DMF, RT, 2 h.

In June of 2020, Gomri et al. prepared new poly(ether pyridine) polymers for the capture of aromatic pollutants such as p-hydroxybenzoic acid, toluic acid, deisopropylatrazine, and 2,4,6-trichlorophenol, along with their halogenated derivatives, in contaminated water sources [73]. To synthesize the beginning monomers, SNAr was again utilized by adding nitrogen- and oxygen-based nucleophiles to PFPy (Scheme 22) [73]. In all cases, the nucleophile was added in the 4-position of PFPy, due to the stabilizing effect of the nitrogen carrying the extra but momentary negative charge, and resulted in the formation of pyridinium-based monomers. 

**Scheme 23:**
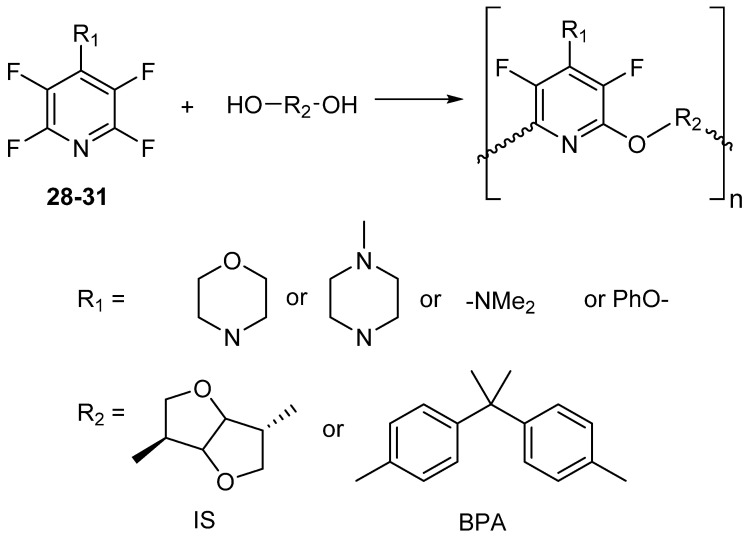
Synthesis of poly(ether pyridine) polymers **P1**–**P8**. Conditions = K_2_CO_3_, DMSO, 24 h.

Once the monomers were obtained, polycondensation reactions with bisphenol A (BPA) and isosorbide (IS) were performed to obtain the poly(ether pyridine) polymers. In order to perform the polycondensation, more forcing conditions were necessary. Both diols, BPA and IS, were heated to 140 °C and 160 °C, respectively (Scheme 23) [73].

The resulting polymers were thermally characterized by DSC and TGA. The polymers had a *T_g_* range of 102 to 206 °C and the 5% decomposition temperature ranged from 346 to 419 °C. The molecular weights determined by GPC did vary depending upon the monomers that were used. The largest molecular weight obtained with IS was evident with monomer **28**, resulting in a number average molecular weight (*M*_n_) of 21,330 g/mol. Were as for BPA, there were two monomers which performed decently in the polymerization, **28** and **30** having *M*_n_ of 105,700 g/mol and 25,170 g/mola, respectively. The dispersity for all the polymers ranged from 1.3 to 4.5 (see Table 8) [73].

The adsorption characteristics of the polymers were evaluated towards a variety of aromatic pollutants and their halogenated derivatives. Polymers based on IS showed improved adsorption capacity towards all the pollutants tested. Furthermore, polymers containing the morpholine-based monomer, **28**, demonstrated enhanced absorption efficiencies greater than 90%. Thus, of all the polymers tested, **28**/IS had the highest efficiency towards the pollutants and their derivatives. The adsorption kinetics were found to fit well to a pseudo-second order mechanism, obeying a Freundlich isotherm model [73].

**Scheme 24:**
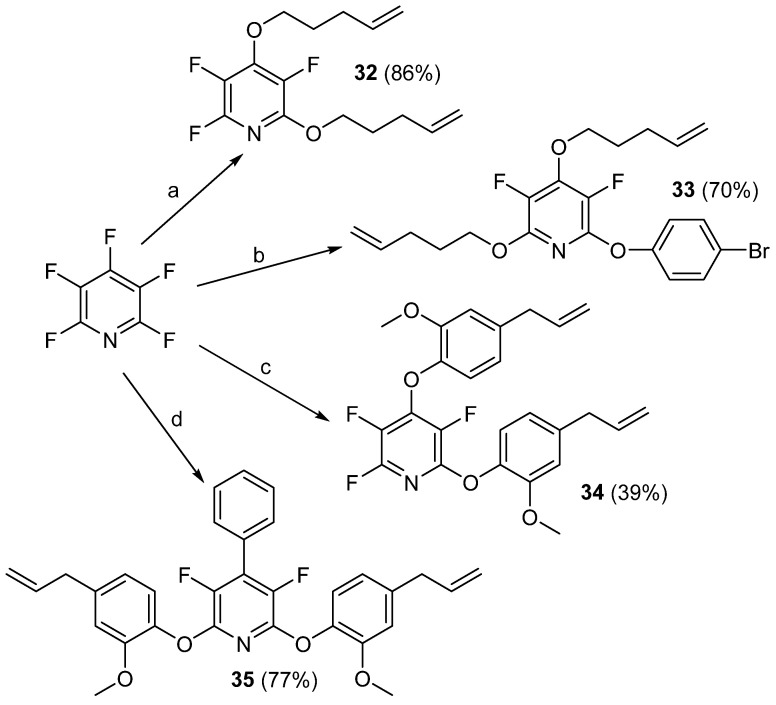
Synthesis of PFPy-based monomers, **32**–**35**. Conditions: a = 4-penten-1-ol, Cs_2_CO_3_, ACN, RT, 72 h; b = from **32**, 4-bromophenol, Cs_2_CO_3_, DMF, 65 °C, 24 h; c = eugenol, Cs_2_CO_3_, DMF, RT, 24 h; d = from **24**, eugenol, Cs_2_CO_3_, DMF, RT, 24 h.

In mid-2021, Stewart and co-workers were able to demonstrate that PFPy could be deployed in high-temperature silicon-based linear and network elastomers and oils [74]. As seen in the other examples above, the authors utilized the regioselective SNAr reaction to prepare a number of new PFPy-based monomers (Scheme 24) [74].

**Scheme 25:**
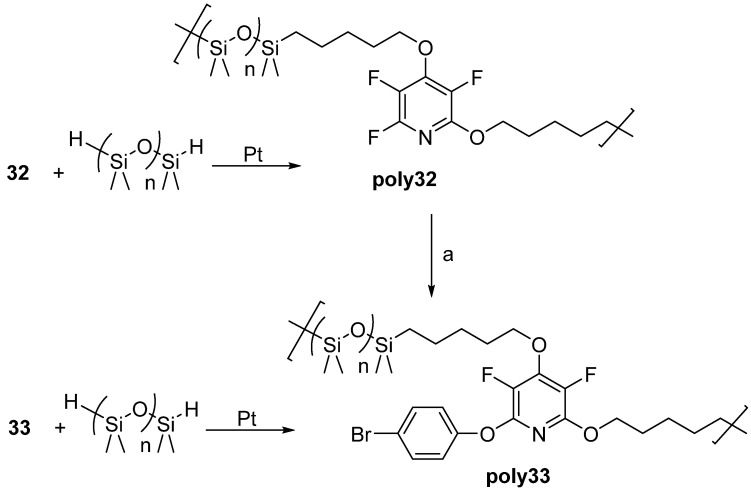
Linear polymers prepared from **32**, **33**, and post-modification of **poly32**. Conditions: a = 4-bromophenol, Cs_2_CO_3_, d_7_-DMF, 60 °C, 72–120 h.

By reacting the terminal alkenes of these monomers with hydride-terminated polydimethylsiloxanes (H-PDMSs), linear silicon-based elastomers and oils were obtained. Interestingly, the polymer made from monomer **32** was found to be post-modifiable with 4-bromophenol to obtain a polymer with similar properties to that of one made from monomer **33** (Scheme 25) [74].

**Figure 4:**
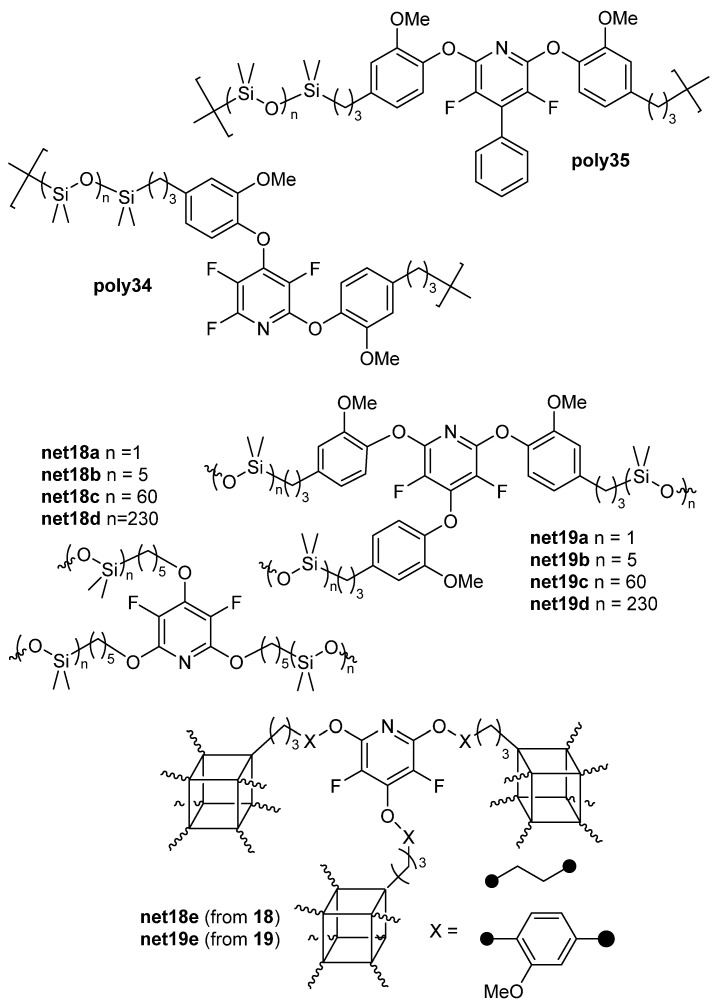
Linear polymers and networks prepared **18**, **19**, **34**, and **35**.

Additionally, compounds **18** and **19** that were previously reported, along with octadimethylhydrosilyl cubic siloxane (OctaSilane POSS), were utilized to form silicon-based networks (Figure 4) [69,74].

The potential application of this work focused on heat-shielding materials (HSMs) in rocket motor casings. These protective coatings not only have to withstand high heat upon re-entry but also extreme cold when deep in space. Thermal characterization showed that the linear polymers made with H-PDMs (**poly32**–**35**) had *T_g_*s ranging from −28 to −12 °C and *T*_d_s ranging from 382 to 404 °C. Network polymers made from H-PDMS (**net18a**–**e** and **net19a**–**e**) were thermally stable with no onset of degradation up to 431 and 430 °C, and char yields as high as 43 and 62%, under inert pyrolysis conditions, respectively. In addition, aliphatic or aromatic content are programmable in order to control glass transition temperatures of the networks, allowing for *T_g_*s ranging from −49 to 83 °C (Table 9) [74].

**Scheme 26:**
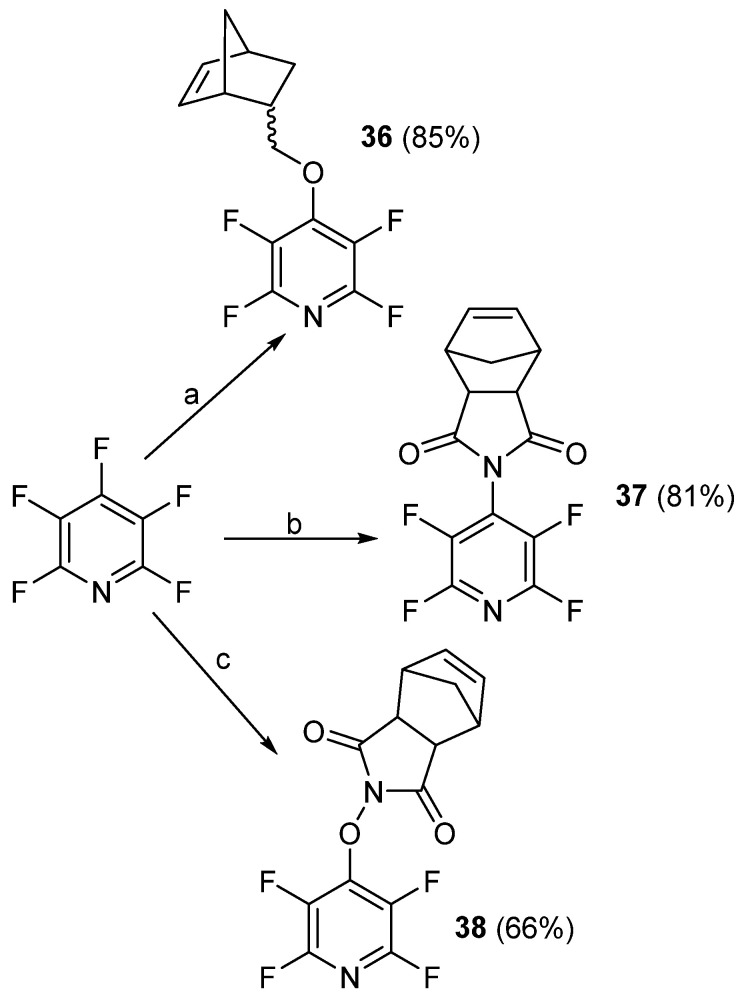
ROMP monomers, **36**–**38**, prepared from PFPy. Conditions: a = bicycle[2.2.1]hept-5-en-2-ylmethanol, Cs_2_CO_3_, THF, RT, 16 h; b = (3a*R*,4*S*,7*R*,7a*S*)-3a,4,7,7a-tetrahydro-1*H*-4,7-methanoisoindole-1,3(2*H*)-dione, Et_3_N, DMF, RT, 24 h; c = (3a*R*,4*S*,7*R*,7a*S*)-2-hydroxy-3a,4,7,7a-tetrahydro-1*H*-4,7-methanoisoindole-1,3(2*H*)-dione, K_2_CO_3_, DMF, RT, 24 h.

Later, in 2021, Houck et al. demonstrated the ability to prepare a variety of aryl ether-functionalized polymers using both pre- and post-functionalized approaches [75]. In order to accomplish this, the authors initially prepared three monomers capable of undergoing ring-opening metathesis polymerization (ROMP) (Scheme 26) [75]. Compounds **37** and **38** had been previously reported [76].

**Scheme 27:**
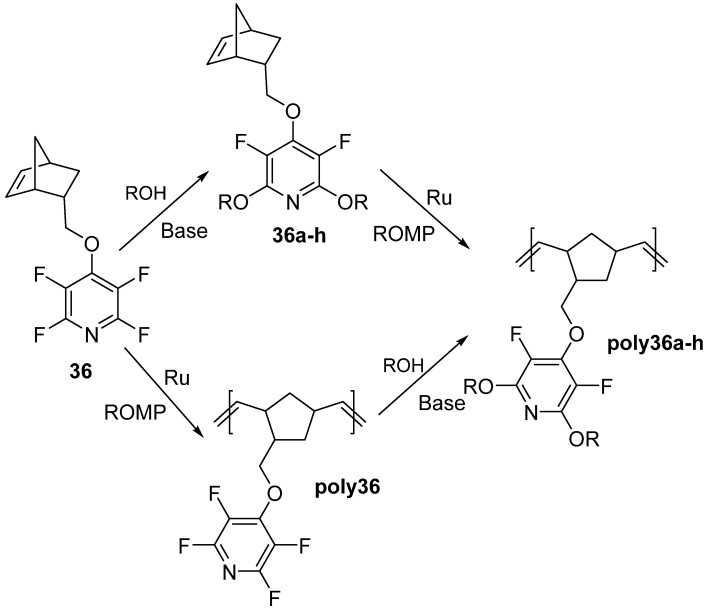
Monomer functionalization and polymer post-functionalization method applied to **36**. ROH: **a** = phenol, **b** = 4-methoxyphenol, **c** = eugenol, **d** = 4-bromophenol, **e** = 4-nitrophenol, **f** = propargyl alcohol, **g** = 4-methoxyphenol and 4-bromophenol, **h** = 2-napthanlenol.

Monomer **36** was then either functionalized with ROH via SNAr or polymerized, to give **poly36**. **Poly36** could then post-functionalized with the ROH nucleophiles (Scheme 27) [75]. 

Similar methods were applied to monomer **37**, but only ROH = **a**–**f** were utilized. Interestingly, this is the first known report of chain growth polymers containing PFPy as a pendant group. Polymers prepared from **36** and **37** were characterized by GPC, TGA, and DSC (Table 10) [75]. 

Attempts to react monomer **38** with ROH resulted in a mixture of products and was attributed to the reversibility sometimes observed in the SNAr reaction at the 4-postion [45,68]. The reversibility was also seen when attempts to post-functionalize **poly38** with the various ROH nucleophiles resulted in densely cross-linked polymer. DFT calculations, along with the experimental observations on the monomers utilized in this study, support that the reversibility of **38** could be attributed to having a small dihedral angle [75]. 

## 5. Conclusions and Perspective

Organofluorine compounds are highly sought after materials because of their unique chemistries and desired properties. With its commercial availability and distinctive reactivity, PFPy is no exception and it has found use in a number of advanced applications. Despite this, it is the authors’ perspective that the use of PFPy in obtaining fluorinated polymers and materials is still gaining momentum. Although its synthesis was first reported in the 1960s, PFPy was not utilized for preparing a fluorinated polymer until nearly 20 years later. To the authors’ knowledge, there are only about 15 peer-reviewed publications with PFPy being employed to make fluorinated networks or fluoropolymers, with the majority of these works being published within the last decade. In all accounts, PFPy is utilized via SNAr to craft the fluorinated network material or polymer, and there is only one example where PFPy is used to fluorinate an existing polymer. Additionally, the majority of these fluoropolymers and networks employ PFPy in the backbone of the polymer versus a pendant group. Lastly, most of these advanced fluorinated materials are obtained through step-growth polymerization techniques, which might be attributed to the propensity of PFPy to react with nucleophiles.

## Figures and Tables

**Table 1:** Reaction summary from Bardin et al. [34].

Entry	Catalyst/co-Catalyst	Conditions	PFPy Yield (%)
1	Fe	500 °C	40–43
2	Zn	THF, 80 °C, 5 h	18
3	Zn/SOCl_2_	Sulfolane, 80 °C, 5 h	30
4	Zn/CuSO_4_	Sulfolane, 80 °C, 5 h	38
5	Zn/CuSO_4_	Diglyme, 80 °C, 5 h	29
6	Zn/complex NiCl_2_x6H_2_O + bpy	DMA, 80 °C, 5 h	34

**Table 2:** Characterization data obtained for **2** and **3 [56]** ^1^.

Polymer	η_inh_ (dL/g)	*T*_g_ (°C)	*T*_d_ (°C, O_2_/Air)
**2 (*n*/m ~ 2)**	0.10	2	265/330
**3 (*n*/m ~ 2)**	0.50	−5	300/350
**3 (*n*/m ~ 2)**	0.22	−12 ^2^	300/360
**3 (*n* = 0, m = 1)**	0.19	−10	310/350

^1^ When data for more than one sample are given, samples are from different experiments.^2^ At 20 °C/min.

**Table 3:** Data obtained by Seyb and Kerres [61].

Polymer/Blend	*M*_n_ (kDa)	*M*_w_ (kDa)	*Ɖ*	*T*_SO3H_ (°C)	*T*_backbone_ (°C)	σ (S/cm)
**8**	12.5	30	2.39	328	332	
**9**	5.9	10.2	1.74	378	366	
**8/PBI-Celazol^®^**				359	461	0.04
**9/PBI-Celazol^®^**				419	410	0.02

**Table 4:** Selected data obtained by Vaganova et al. [64].

PI/co-PI	*M*_n_ (kDa)	η_inh_ (dL/g)	*T*_g_ (°C)	*T*_5%_ ^1^ (°C)	*T*_5%_ ^2^ (°C)
PI **10**	14.5	0.09	345	515	493
PI **11**	17.5	-	347	519	491
PI **12**	30.5	0.18	346	519	503
PI **13**	12.0	0.14	344	504	500
PI **14**	22.5	0.17	315	432	430
co-PI **11**	ND	0.45	300	526	513
co-PI **12**	ND	0.42	288	525	495
co-PI **13**	ND	0.35	284	522	507
co-PI **14**	ND	0.28	294	465	480

^1^ Temperature at 5% weight loss under He. ^2^ Temperature at 5% weight loss under a 80:20 He:O_2_ mixture.

**Table 5:** Selected thermal data obtained by Moore and co-workers [69].

System ^1^	*T*_g_ (°C)	Predicted *T*_g_^2^ (°C)	*T*_d,5%_ (°C)	Char Yield (%)
**18**			143	0
**19**			259	7.5
ODT				
100:0	−42	-	274	13.2
75:25	−29	−28	279	17.8
50:50	−15	−16	289	21.7
25:75	−3	−6	296	23.3
0:100	3	-	325	23.2
TMPMP				
100:0	−17	-	275	13.2
75:25	1	−5	280	18.8
50:50	10	5	288	23.5
25:75	19	14	305	23.5
0:100	21	-	348	28.7

^1^ Ratios given are mol ratios of **18**:**19** used in the thiol-ene network with either ODT or TMPMP, respectively. ^2^ Predicted *T_g_* value obtained from the Fox equation.

**Table 6:** Selected thermal data obtained by Houck et al. [70].

Entry	*T*_m_ (°C)	*T*_onset_ (°C)	*T*_max_ (°C)	*T*_d_ Ar/Air (°C)	Char Yield ^1^ Ar/Air (%)
**22**	93, 108	306	341	470/480	51/2
**23**	-	327	372	456/483	81/1

^1^ Determined at 900 °C.

**Table 7:** Selected thermal data obtained by Eismeier and co-workers [71].

Network	*T*_g_^1^ (°C)	*T*_d_^2^ (°C)	Char Yield ^2^ (%)
**26**	44	100	45
**27**	140	210	58

^1^ Under nitrogen. ^2^ Under argon.

**Table 8:** Selected thermal and molecular weight data obtained by Gomri et al. [73].

Polymer	Yield ^1^ (%)	*T*_g_^2^ (°C)	*T*_d_^3^ (°C)	*M*_n_^4^ (g/mol)	*M*_w_^4^ (g/mol)	*Ɖ* ^4^
**28**/BPA	98	146	419	105,700	291,700	2.7
**28**/IS	92	206	365	21,330	49,600	2.3
**29**/BPA	73	127	395	1961	2871	1.4
**29**/IS	79	170	363	1800	2454	1.3
**30**/BPA	95	124	408	25,170	112,000	4.5
**30**/IS	71	143	357	2743	4462	1.6
**31**/BPA	88	102	444	3523	10,590	4.1
**31**/IS	91	155	346	3017	6161	2

^1^ Precipitation into MeOH, evaporation under vacuum at 60 °C. ^2^ Obtained by DSC with heating at 10 °C/min. ^3^ Temperature at 5% mass loss. ^4^ Determined by GPC in CHCl_3_ against PS standards.

**Table 9:** Selected thermal data obtained by Stewart and co-workers [74].

Polymer/Network	*T*_g_^1^ (°C)	*T*_d_^5^ (°C)	Char Yield (%)
poly**32**	−25	360	3
poly**33**	−12	404	2
poly**34**	−26	388	14
poly**35**	−28	382	13
net**18a ^2^**	−45	378	9
net**18b ^2^**	−43	379	18
net**18c ^2^**	−49	409	43
net**18d ^2^**	- ^3^	418	33
net**18e ^2^**	28	407	49
net**19a ^2^**			
net**19b ^2^**	10	402	34
net**19c ^2^**		409	25
net**19d ^2^**	- ^4^	431	30
net**19e ^2^**	83	410	62

^1^ DSC in nitrogen with heating at 5 °C/min determined on the third heating cycle. ^2^ Data obtained after Soxhlet extraction. ^3^ Melt transition observed at −46 °C. ^4^ Melt transition observed at −38 °C. ^5^ MTGA onset in nitrogen with heating at 10 °C/min.

**Table 10:** Selected characterization data for the polymers prepared by the pre- and [post-] functionalization methods developed by Houck et al. [75].

Polymer	*M*_w_^1^ (kDa)	*Ɖ* ^4^	*T*_g_^2^ (°C)	*T*_d_^3^ (°C)	Char Yield ^4^ (%)
poly**36**	332.7	2.5	6	127	8
poly**36a**	73.7 [128.9]	1.9 [1.4]	62	192	33
poly**36b**	112.1 [275.1]	2.1 [2.0]	62	247	28
poly**36c**	[228.1]	2.8		153	40
poly**36d**	32.1 [171.7]	2.1 [2.6]	138	312	9
poly**36e**	[54.9]	[2.2]	77	265	50
poly**36f**	[22.9]	[2.1]		181 (405) ^5^	45 (67) ^5^
poly**36g**	142.4	3.5	85	250	38
poly**36h**	[172.3]	[2.8]	81	293	29
poly**37**	217.0	2.1	227	229	30
poly**37a**	55.4 [28.2]	1.9 [2.0]	186	392	29
poly**37b**	192.7 [55.5]	2.0 [1.8]	175	385	35
poly**37c**	2.3	2.8	100	376	58
poly**37d**	69.9	1.9	190	368	26
poly**37e**	127.0	3.2		233	52
Poly**37f**	[73.3]	[1.8]		62 (355) ^5^	49 (60) ^5^

^1^ Determined by GPC in THF against PS standards. ^2^ DSC in nitrogen with heating at 5 °C/min, determined on the third heating cycle. ^3^ TGA onset in nitrogen with heating at 10 °C/min. ^4^ TGA at 900 °C in nitrogen. ^5^ Values observed as the second scan after cure by DSC.

## Data Availability

Not applicable.
